# The prognostic impact of tumor length in esophageal cancer: Protocol for a systematic review and meta-analysis

**DOI:** 10.1097/MD.0000000000012902

**Published:** 2018-10-26

**Authors:** Xiangwei Zhang, Yang Wang, Yuanzhu Jiang, Zhaoyang Wang, Linping Zhao, Xianbiao Xue, Shaowei Sang, Lin Zhang

**Affiliations:** a Department of Thoracic Surgery; b Department of Medical Imaging, Shandong Provincial Hospital Affiliated to Shandong University, Shandong University, Jinan; c Department of Thoracic Surgery, Shouguang City People's Hospital, Shouguang; d Department of Thoracic Surgery, Juye County People's Hospital, Juye; e Clinical Epidemiology Unit, Qilu Hospital of Shandong University, Jinan, PR China.

**Keywords:** esophageal cancer, meta-analysis, prognosis, systematic review, tumor length

## Abstract

**Background:**

More and more studies were performed to explore the prognostic role of tumor length in esophageal cancer (EC). However, the results remain controversial. Hence, the aim of the review was to evaluate the association between tumor length and oncologic outcome in EC patients through meta-analysis.

**Methods:**

A systematic literature search for relevant articles published in English language will be conducted in the PubMed, Web of Science, and Embase. Hazard ratio and 95% confidence intervals (CIs) will be employed as effect measures to estimate the correlation between tumor length and the oncologic outcomes including overall survival, disease-free survival, progression-free survival, relapse-free survival, and cancer-specific survival. We will use the software STATA 14.0 to perform the meta-analysis to calculate the data synthesis.

**Results:**

The review will provide a high-quality synthesis of current evidence of the prognostic role of tumor length in ECs. The results will be published in a peer-reviewed journal.

**Conclusion:**

This will be the first systematic review and meta-analysis to evaluate the prognostic role of tumor length in EC patients. The results will better predict EC survival and identify higher-risk patients for postoperative therapy.

**PROSPERO registration number:**

This systematic review protocol has been registered in the PROSPERO network (No. CRD42018106851).

## 1 Introduction

Esophageal cancer (EC) is one of the most common and lethal cancers worldwide. The majority of patients die within 1 year of diagnosis, and only 8% to 20% of patients are alive at 5 years.^[1]^ Strategies to improve the outcome of EC have focused on multimodality treatment, including surgery, chemotherapy, and radiotherapy.^[2,3]^ Although current practice incorporating chemotherapy or radiation into the treatment protocol, surgical resection may remain the only chance for curing this disease. Complete surgical resection with radical lymphadenectomy also provides accurate staging information, which is important for outcome prediction and treatment decision making. An effective and rational staging system of EC is the essential prerequisite to determine the appropriate treatments and predict long-term survival.^[4]^

The confirmed prognostic indicators include histological grading, tumor differentiation, tumor (T), nodal metastases (N), and metastasis (M) classification, whereas the current TNM classification system does not consider the tumor length in the staging and classification scheme for patients with esophageal carcinoma.^[5,6]^ In recent years, more and more studies have observed that tumor length was an independent prognostic predictor. Bolton et al^[7]^ analysis 133 patients with EC who received surgery resection and found tumor length was a risk factor for long-term survival and lymph node involvement in early stage patients. Wang et al^[8]^ retrospectively reviewed 582 patients with EC who underwent curative resection and demonstrated that tumor length could have a significant impact on the overall survival (OS) and disease-free survival (DFS). Our early studies implied that tumor length was an important prognostic factor on both the OS and cancer-specific survival (CSS).^[9,10]^ However, the prognostic role of tumor length in EC is still controversial. Kahn et al^[11]^ indicated that tumor length was not a prognostic factor in N0 EC patients. Hence, the aim of this study was to use meta-analysis to evaluate the prognostic value of tumor length for survival in patients with EC patients. To the best of our knowledge, this is the first meta-analysis to investigate the prognostic role of in patients with EC.

## 2 Methods

### 2.1 Study registration

The Preferred Reporting Items for Systematic Reviews and Meta-Analyses Protocols (PRISMA-P) statement guidelines will be followed in the protocol.^[12]^ This systematic review and meta-analysis has been registered on PROSPERO with registration number: CRD CRD42018106851. Ethical approval is not required because this is a study based on aggregate data and did not involve humans.

#### 2.1.1 Data sources and search strategy

The PubMed, Embase, and Web of Science databases will be searched for relevant articles. Both full text and MeSH search for keywords were used. The reference lists and related articles in each identified publication were also reviewed for potential studies. In addition, the reference lists of relevant publication were also reviewed to avoid missing relevant studies.

Search strategy of PubMed was as follows: (tumor length) OR (tumor size); (esophageal neoplasm) OR (esophageal cancer) OR (esophageal carcinoma) OR (EC); (prognosis) OR (survival); Step 1 AND step 2 AND step 3.

### 2.2 Inclusion and exclusion criteria

Articles will be included if they meet the following criteria: retrospective or prospective studies; exploring the relationship between tumor length and clinicopathological features or prognosis of EC; hazard ratios (HRs) and 95% CIs for the associations between tumor length and survival outcomes [OS and DFS/progression-free survival (PFS)/relapse-free survival (RFS)/CSS] were reported; and studies published in English.

Articles will be excluded if they meet the following criteria: abstracts, letters, editorials, reviews, or case reports; studies were not available in English; studies had overlapping or repeat data; studies concerned non-human or non-clinical research; and studies did not present the cut-off value for tumor length.

### 2.3 Data extraction and quality assessment

#### 2.3.1 Selection of studies

**Figure 1:**
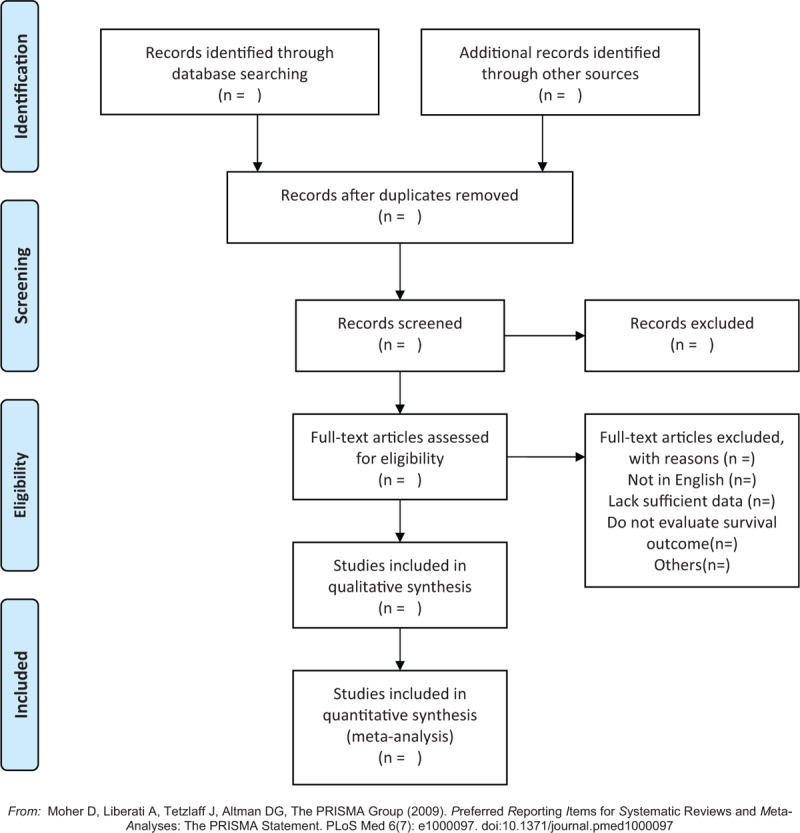
Flow chat of literature search and selection.

Two reviewers (XZ and YW) will select the included studies and extract relevant data independently from the studies. Any disagreement will be solved by group discussion. If necessary, the third reviewer (YJ) will be consulted. The selection process will be summarized according to PRISMA flow diagram (Fig. 1).

#### 2.3.2 Data extraction and management

Two researchers (XZ and YW) reviewed the eligible articles independently. Any disagreement will be solved by consensus or an arbiter (YJ). HRs were extracted preferentially from multivariable analyses where available. Otherwise, HRs from univariate analyses were extracted. The data were obtained directly from individual articles or were calculated from indirect data.^[13]^ In addition, other clinic pathologic parameters were extracted using a unified form including author, year of publication, country of origin, total number of patients, age, sex, follow-up time, operation strategy, TNM stage, and cut-off value of tumor length.

#### 2.3.3 Assessment of quality in included studies

The quality of the included studies was assessed through the Newcastle-Ottawa Quality Assessment Scale (NOS) for no randomized studies, which consists of 3 parts: selection (0–4 points), comparability (0–2 points), and outcome assessment (0–3 points). The maximum score is 9 points and NOS scores ≥7 were assigned as high-quality studies.^[14]^ Two reviewers (XZ and YW) will independently assess the quality of each study and any conflicts disagreements in quality assessment were resolved by joint discussion.

#### 2.3.4 Measures of prognosis

Pooled HRs and 95% CIs were used to analyze the relationship between tumor length and prognosis (OS or DFS/PFS/RFS/CSS). HRs were extracted preferentially from multivariable analyses where available. Otherwise, HRs from univariate analyses were extracted. The data were obtained directly from individual articles or were calculated from indirect data using the methods proposed by Tierney et al.^[13]^

#### 2.3.5 Management of missing data

Some data are missing in several included studies, so we will contact the corresponding author to request any inadequate and missing data by E-mail. If the data are still incomplete, we will perform data synthesis through available information and address the potential impact of missing data on the pooled results in the discussion parts.

#### 2.3.6 Assessment of heterogeneity

Assessment of heterogeneity between the included studies will be conducted to evaluate the feasibility of meta-analysis. The Cochran Q test and Higgins *I*^2^ method will be used to assess the heterogeneity.^[15]^ A *P* < .05 for Q test or *I*^2^ > 50% for *I*^2^ test suggested significant heterogeneity in the literature, whereas a *P* > .05 for *Q* test or *I*^2^ < 50% for *I*^2^ test indicated no heterogeneity. In cases of substantial heterogeneity, we will perform subgroup analysis to explore the potential causes.

#### 2.3.7 Data synthesis

All the statistical analyses will be performed using STATA statistical software version 14.0 (STATA, College Station, TX). The pooled HR and 95% CIs will be used to assess the prognostic role of tumor length in EC patients. A *P* < .10 for *Q* test or *I*^2^ > 50% for *I*^2^ test suggested significant heterogeneity in the literature and a random-effect model (DerSimonian-Laird method) was used.^[16]^ Otherwise, the fixed-effects model (Mantel-Haenszel method) was adopted.^[17]^ In addition, subgroup analysis and sensitivity analysis will also be conducted to explain the possible heterogeneity. All *P* values were 2 sided. A *P* < .05 was considered statistically significant.

#### 2.3.8 Subgroup analysis

If the necessary data are available, subgroup analysis will be conducted to determine the possible factors that may influence the results: different nationality of patients; different histology type of EC; different treatment strategies; different statistical analysis methods; and different cut-off values.

#### 2.3.9 Sensitivity analysis

Sensitivity analysis will be conducted by omitting each single study every time to see the influence of the individual dataset on the pooled HRs. The results will not be substantially changed when any study is excluded if the pooled HRs are robust.

#### 2.3.10 Publication bias

Begg funnel plot and Egger test linear regression test will be conducted to evaluate the publication bias of the included studies.^[18,19]^ If publication bias is detected (*P* < .05 is considered statistically significant), we will perform the “trim and fill” test for further analysis.

## 3 Discussion

EC is one of the deadliest malignances worldwide with a poor 5-year survival rate of approximately 20%.^[1]^ With improvements in the treatment techniques, the strategies have focused on multimodality treatment, including surgery, chemotherapy, and radiotherapy. Complete surgical resection with radical lymphadenectomy could provide accurate TNM staging information. The rational staging system for EC is critical to determine the appropriate management and predict long-term survival.^[4]^ In the latest American Joint Committee on Cancer staging system for EC, histological grading and tumor location as well as depth of esophageal wall invasion (T classification), lymph node involvement (N classification), and distant metastasis (M classification) are regarded as prognostic factors.^[5]^ However, heterogeneous clinical courses are frequently observed even within the same tumor stage. So many other clinicopathological factors predicting the outcome of cancer patient have been investigated over the past decades.^[20,21]^

Nowadays more and more studies have focused the prognostic role of tumor length in EC patients. Unfortunately, these results still remain controversial. Wang et al^[8]^ evaluated 582 patients with esophageal squamous cell carcinoma who underwent surgical resection and indicated that tumor length could impact the OS. Tumor length may provide additional prognostic information to the current TNM staging system. Yendamuri et al^[22]^ and Song et al^[23]^ demonstrated similar results. However, Kahn et al^[11]^ indicated that tumor length was not a prognostic factor in N0 EC patients. To better understand the impact of tumor length on survival in EC patients, we conducted the meta-analysis to provide more objective and accurate proofs of the relations between tumor length and the prognosis of patients with EC.

Several limitations need to be addressed in our review. Firstly, only studies published in English will be included which may cause the publication bias in our study. Secondly, different resources of patients and pathological types, different treatment strategies, and duration of follow-up, different cut-off value of tumor length and statistic methods, will increase the heterogeneity in our study. Although we will conduct Begg funnel plot and Egger test to evaluate the publication bias and undertake the subgroup analysis to explain the heterogeneity, these limitations may hinder the application of the ratios in the clinical work. Further meta-analyses including additional studies and increased sample sizes are needed to correct for publication bias and heterogeneity and improve the accuracy.

## Author contributions

Xiangwei Zhang: Funding acquisition; Methodology; Writing – original draft

Yang Wang: Conceptualization; Data curation; Formal analysis; Writing – original draft

Yuanzhu Jiang: Formal analysis; Methodology; Software

Zhaoyang Wang: Investigation; Software; Validation

Linping Zhao: Data curation; Investigation; Methodology

Xianbiao Xue Data curation; Investigation; Methodology

Shaowei Sang Formal analysis; Software; Writing – review and editing

Lin Zhang: Funding acquisition; Writing – review and editing

**Conceptualization:** Yang Wang.

**Data curation:** Yang Wang, Linping Zhao, Xianbiao Xue.

**Formal analysis:** Yang Wang, Yuanzhu Jiang, Shaowei Sang.

**Funding acquisition:** Xiangwei Zhang, Lin Zhang.

**Investigation:** Zhaoyang Wang, Linping Zhao, Xianbiao Xue.

**Methodology:** Xiangwei Zhang, Yuanzhu Jiang, Linping Zhao, Xianbiao Xue.

**Software:** Yuanzhu Jiang, Zhaoyang Wang, Shaowei Sang.

**Validation:** Zhaoyang Wang.

**Writing – original draft:** Xiangwei Zhang, Yang Wang.

**Writing – review and editing:** Shaowei Sang, Lin Zhang.

## References

[1] Siegel RL Miller KD Jemal A Cancer Statistics, 2017. CA Cancer J Clin 2017;67:7–30. 28055103 10.3322/caac.21387

[2] Chiu PW Chan AC Leung SF Multicenter prospective randomized trial comparing standard esophagectomy with chemoradiotherapy for treatment of squamous esophageal cancer: early results from the Chinese University Research Group for Esophageal Cancer (CURE). J Gastrointest Surg 2005;9:794–802. 16187480 10.1016/j.gassur.2005.05.005

[3] Blazeby JM Strong S Donovan JL Feasibility RCT of definitive chemoradiotherapy or chemotherapy and surgery for oesophageal squamous cell cancer. Br J Cancer 2014;111:234–40. 24921919 10.1038/bjc.2014.313 PMC4102950

[4] Kato H Nakajima M Treatments for esophageal cancer: a review. Gen Thorac Cardiovasc Surg 2013;61:330–5. 23568356 10.1007/s11748-013-0246-0

[5] Rice TW Esophageal cancer staging. Korean J Thorac Cardiovasc Surg 2015;48:157–63. 26078921 10.5090/kjtcs.2015.48.3.157 PMC4463223

[6] Rami-Porta R Bolejack V Crowley J The IASLC lung cancer staging project: proposals for the revisions of the T descriptors in the forthcoming eighth edition of the TNM classification for lung cancer. J Thorac Oncol 2015;10:990–1003. 26134221 10.1097/JTO.0000000000000559

[7] Bolton WD Hofstetter WL Francis AM Impact of tumor length on long-term survival of pT1 esophageal adenocarcinoma. J Thorac Cardiovasc Surg 2009;138:831–6. 19660349 10.1016/j.jtcvs.2009.02.003

[8] Wang BY Goan YG Hsu PK Tumor length as a prognostic factor in esophageal squamous cell carcinoma. Ann Thorac Surg 2011;91:887–93. 21353021 10.1016/j.athoracsur.2010.11.011

[9] Zhang X Wang Y Li C The prognostic value of tumor length to resectable esophageal squamous cell carcinoma: a retrospective study. PeerJ 2017;5:e2943. 28168111 10.7717/peerj.2943 PMC5289103

[10] Zhang X Wang Y Qu P Prognostic value of tumor length for cause-specific death in resectable esophageal cancer. Ann Thorac Surg 2018;106:1038–46. 29883640 10.1016/j.athoracsur.2018.05.018

[11] Khan OA Alexiou C Soomro I Pathological determinants of survival in node-negative oesophageal cancer. Br J Surg 2004;91:1586–91. 15505868 10.1002/bjs.4778

[12] Shamseer L Moher D Clarke M Preferred reporting items for systematic review and meta-analysis protocols (PRISMA-P) 2015: elaboration and explanation. BMJ 2015;350:g7647. 25555855 10.1136/bmj.g7647

[13] Tierney JF Stewart LA Ghersi D Practical methods for incorporating summary time-to-event data into meta-analysis. Trials 2007;8:16. 17555582 10.1186/1745-6215-8-16 PMC1920534

[14] Goebell PJ Kamat AM Sylvester RJ Assessing the quality of studies on the diagnostic accuracy of tumor markers. Urol Oncol 2014;32:1051–60. 25159014 10.1016/j.urolonc.2013.10.003 PMC4524775

[15] Higgins JP Thompson SG Deeks JJ Measuring inconsistency in meta-analyses. BMJ 2003;327:557–60. 12958120 10.1136/bmj.327.7414.557 PMC192859

[16] DerSimonian R Laird N Meta-analysis in clinical trials. Control Clin Trials 1986;7:177–88. 3802833 10.1016/0197-2456(86)90046-2

[17] Mantel N Haenszel W Statistical aspects of the analysis of data from retrospective studies of disease. J Natl Cancer Inst 1959;22:719–48. 13655060

[18] Begg CB Mazumdar M Operating characteristics of a rank correlation test for publication bias. Biometrics 1994;50:1088–101. 7786990

[19] Egger M Davey Smith G Schneider M Bias in meta-analysis detected by a simple, graphical test. BMJ 1997;315:629–34. 9310563 10.1136/bmj.315.7109.629 PMC2127453

[20] Wei C Deng WY Li N Lymph node ratio as an alternative to the number of metastatic lymph nodes for the prediction of esophageal carcinoma patient survival. Dig Dis Sci 2015;60:2771–6. 25939544 10.1007/s10620-015-3681-1

[21] Tachezy M Tiebel AK Gebauer F Prognostic impact of perineural, blood and lymph vessel invasion for esophageal cancer. Histol Histopathol 2014;29:1467–75. 24819775 10.14670/HH-29.1467

[22] Yendamuri S Swisher SG Correa AM Esophageal tumor length is independently associated with long-term survival. Cancer 2009;115:508–16. 19117343 10.1002/cncr.24062

[23] Song Z Wang J Lin B Analysis of the tumor length and other prognosis factors in pT1-2 node-negative esophageal squamous cell carcinoma in a Chinese population. World J Surg Oncol 2012;10:273. 23249675 10.1186/1477-7819-10-273 PMC3560067

